# An Extremely Rare Case of Lower Urinary Tract Symptoms: Floating Benign Mesenchymal Mass in Abdomen

**DOI:** 10.1155/2017/9854343

**Published:** 2017-02-23

**Authors:** Bulent Kati, Yigit Akin, Eyyup Sabri Pelit, Mehmet Ogur Yilmaz

**Affiliations:** ^1^Department of Urology, Faculty of Medicine, Harran University, Sanliurfa, Turkey; ^2^Department of Urology, Faculty of Medicine, Izmir Katip Celebi University, Izmir, Turkey

## Abstract

A 48-year-old man admitted to the urology outpatient clinic with major symptoms of right-side pain and intermittent lower urinary tract symptoms (LUTSs) such as low urine flow rate, dysuria, and frequency. Uroflowmetry showed low urine flow, and laboratory tests revealed no pathology. Ultrasound (US) showed a 7 cm calcific mass above the bladder and a kidney cyst with a diameter of 5.3 cm in the upper pole of the right kidney. Enhanced computed tomography confirmed the US findings. Laparoscopic transperitoneal* renal* cyst decortication was performed. There was no sign of additional tumors. An independent mass in the abdomen was diagnosed, and the mass was removed. Based on the pathology, the diagnosis was a benign mesenchymal calcific mass. This is the first report of LUTSs due to a free benign mesenchymal mass in the published literature.

## 1. Introduction

Lower urinary tract symptoms (LUTSs) are a frequent complaint in men older than 45 years [1]. Such complaints raise a suspicion of benign prostatic hyperplasia. However, cases of lower bladder capacity require further clinical investigations.

A benign mesenchymoma is a rare soft tissue tumor that consists of fibrous tissue and two or more differentiated types of cells of mesenchyme origin that are not found at the host site. Benign mesenchymomas may be the result of proliferation of pluripotent primitive mesenchyme cells, which undergo differentiation into various elements [2]. They are most commonly found in extremities, kidneys, trunks, and perirenal areas. The majority are found in the first decade of life. When analyzing the type of tumor, it is important to consider the location of the mass and the morphology of the cells. These two features allow a tumor of mesenchyme origin to be differentiated from other masses, such as hamartomas, choristomas, and teratomas. Abdominal mesenchymal tumors are rare but can cause some clinical complaints [3]. Most are located in the gastrointestinal tract. However, in rare cases, a mesenchymal tumor can be located as a free mass in the abdomen.

We describe an extremely rare case of LUTSs due to a benign mesenchymal mass in the abdomen. According to a literature search, this is the first case of LUTSs associated with a free mesenchymal mass in the abdomen. The mass was removed using laparoscopic surgery.

## 2. Case Presentation

**Figure 1:**
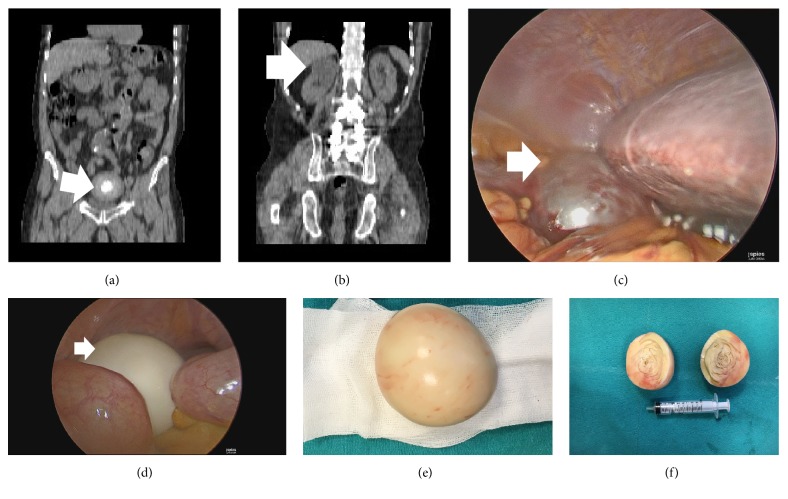
The calcific mass floating independently in abdomen and right kidney mass were diagnosed. (a) Computed tomography revealed a calcific mass with 7 cm diameter that was making pressure on bladder. (b) There was right kidney cyst with 5.3 cm diameter on the upper pole in computed tomography scan. (c) The kidney cyst was decorticated during laparoscopic surgery. (d) The free mass was observed simultaneously in laparoscopic surgery. (e) The mass was taken out directly; there was no attachment around other tissues. (f) The mass was divided into half; it was very rigid.

A 48-year-old man was admitted to the urology outpatient clinic with chief symptoms of right-lumbar pain and intermittent lower urinary tract symptoms (LUTSs) such as low urine flow rate, dysuria, and frequency. There was not any symptom related to pressure to rectum. He had no comorbidities and had not undergone surgery in the past. He reported that he had used nonsteroidal anti-inflammatory medication for right flank pain, but that the pain had not disappeared. Laboratory examinations did not show any abnormality in terms of prostate specific antigen (1.3 ng/dL) or serum creatinine levels (1 mg/dL). Erythrocytes and leukocytes were detected in a urine analysis. On uroflowmetry, the maximum flow rate was 21 mL/min, the average flow rate was 18 mL/min, and the voided volume was 221 mL. Ultrasound (US) showed a simple renal cyst, with a diameter of 5.3 cm on the upper pole of the right kidney and a 7 cm independent calcific mass above the bladder. The calcific mass was compressing the bladder. The position of the abdominal mass did not change while the patient moved during the US examination. Enhanced computed tomography (CT) supported the US findings (Figures 1(a) and 1(b)). No contrast medium was present on the wall of the right renal cyst.

As* Echinococcus granulosus *is common in Southeastern Turkey, an immunofluorescence assay was performed to check the patient's blood for antigens. In addition, a check of the liver revealed no cysts caused by* Echinococcus granulosus*. Furthermore, there was no sign of any malignancy or metastasis from/to the abdominal mass. In view of these findings, the patient signed a consent form and underwent laparoscopic surgery to remove the right renal cyst and calcific mass.

The patient was prepared for laparoscopy. After uncovering the kidney cyst, blunt and sharp dissections were continued up to normal renal parenchyma (Figure 1(c)). The wall of the cyst was then cut, and its contents were aspirated. The roof of the cyst was transected, and a specimen was taken for pathology examinations. A bipolar dissector provided hemostasis. The patient was then placed in a supine, 20-degree Trendelenburg position. However, radiology reported that the mass did not change its place during movements; a free-floating white colored mass was observed in the small intestine above the bladder during laparoscopy (Figure 1(d)). The optic trocar point was expanded, and the mass was easily removed because of its unattached independent origin (Figures 1(e) and 1(f)). We have seen that it has acquired a place in itself that has been comfortably placed only because of its own weight. At the end of the operation, a drainage tube was placed in the area of the renal cyst. The drainage tube was removed on the day after the surgery, and the patient was discharged. The symptoms recovered after surgery in follow-up.

**Figure 2:**
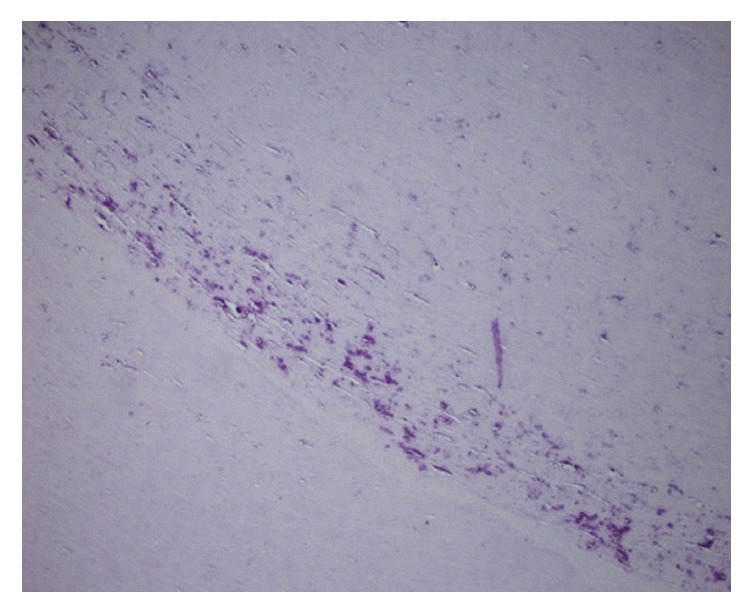
Benign mesenchymal cells and inflammatory elements with a few calcifications periphery of the material are remarkable (H&E ×100).

Pathological examinations revealed a benign renal cyst and benign mesenchymal mass, with an extensive hyalinized area, focal calcifications, and little cellularity on hematoxylin-eosin (H&E) staining. The presence of benign mesenchymal cells and inflammatory elements, with little calcification present on the peripheral side of the mass, is remarkable (Figure 2, H&E ×100).

## 3. Discussion

We described an extremely rare case of LUTSs caused by a free benign mesenchymal mass in the abdomen pressing on the bladder. The mass was removed by laparoscopic route, and decortication of the cyst on the right kidney was performed at the same time. To the best of our knowledge, this is the first case report in the literature of this kind of mass causing LUTSs.

Independent-looking masses could occur in women after a hysterectomy and/or uterus surgery due to residual mesenchymal tissues [3]. Some masses might be caused by parasites or can mimic tumors, notably in the liver [4]. However, in the present case, the patient was male, and a CT examination revealed no sign of any parasitic cysts in any organs. Mesenchymal masses of gastrointestinal origin have been reported in the abdomen. The mass in the present case was independent. In view of the above-mentioned differential diagnoses of mesenchymal masses, we decided to remove the mass by laparoscopic surgery.

Today, the superiority of laparoscopy is generally accepted [5]. In the present case, laparoscopy enabled a minimally invasive approach and simultaneous removal of the kidney cyst and abdominal mass. However, due to the large size of the mass, the optic trocar entry had to be expanded to remove the mass.

Although previous studies reported calcific fibrous masses in the gastrointestinal tract [6] and inflammatory myofibroblastic tumors [7], there have been no published reports of a free mesenchymal mass in the abdomen, notably in men. The pathological findings of the mass in the present case were very different from those reported in the literature, with little cellularity and much hyalinization [6, 7]. In addition, mesenchymal cells only were present on the peripheral side of the mass. Our pathology slides are dyed with H&E. Due to the lack of cellularity in the pathology specimen, the pathologist could not perform an immunohistochemical analysis.

Favorito et al. described the case of a 22-year-old male with hydronephrosis associated with abdominal cryptorchid testis [8]. The patient presented with a hard and painful mass (diameter of approximately 8 cm) in the hypogastric region. After laparoscopic resection of the mass, a histopathological study showed a classic seminoma. There are no reports in the literature of a free abdominal mass above the bladder.

Positron emission tomography can be used rather than CT to detect potential metastases from/to an active malignant mass. In the present case, due to the lack of cellularity in the mass, nothing suggested metastasis, and positron emission tomography was not conducted.

In conclusion, a mesenchymal free-floating mass originating in the abdomen can give rise to LUTSs. When close to or above the bladder, such masses can exert greater effects than other abdominal free masses. Laparoscopy should be the preferred surgical approach in surgical treatment of rare abdominal masses, followed by additional renal pathology.
